# Impact of Drying Regimes and Different Coating Layers on Carboxymethyl Cellulose Cross-Linked with Citric Acid on Cotton Thread Fibers for Wound Dressing Modification

**DOI:** 10.3390/polym14061217

**Published:** 2022-03-17

**Authors:** Mohamad Khalid Khairunnisa-Atiqah, Kushairi Mohd Salleh, A. H. Ainul Hafiza, Nyak Syazwani Nyak Mazlan, Marhaini Mostapha, Sarani Zakaria

**Affiliations:** 1 Bioresources and Biorefinery Laboratory, Faculty of Science and Technology, Universiti Kebangsaan Malaysia, Bangi 43600, Selangor, Malaysia; kat.atiq@gmail.com (M.K.K.-A.); ainulhafiza@uitm.edu.my (A.H.A.H.); nyaksyazwani@gmail.com (N.S.N.M.); 2 Centre of Foundation Studies, Universiti Teknologi MARA, Cawangan Selangor, Kampus Dengkil, Dengkil 43800, Selangor, Malaysia; 3 Centre for Biofuel and Biochemical Research, Institute of Self-Sustainable Building, Universiti Teknologi Petronas, Seri Iskandar 32610, Perak, Malaysia; marhainimostapha@gmail.com

**Keywords:** carboxymethyl cellulose, drying effects, wound dressing, coating layers, fiber-based material, mechanical properties

## Abstract

The oldest preservation techniques used are drying techniques, which are employed to remove moisture and prevent microorganisms’ growths, prolonging a material’s shelf life. This study evaluates the effects of drying methods on carboxymethyl cellulose (CMC) + citric acid (CA) coating layers on cotton threads. For this reason, cotton threads were washed and then coated with different layers of CMC cross-linked with CA, followed by drying using an oven (OD), infrared (IR), and a combination of oven + IR (OIR) drying methods at 65 °C. Our investigations revealed that CMC + CA yields a pliable biopolymer. The differences in drying regimes and coating layers of CMC + CA have a significant effect on the coated cotton thread strength and absorption capability. The study concluded that the IR drying regime is more effective to dry a single-layered cotton thread with a single layer of CMC + CA coating to enhance desirable properties for wound dressing modification.

## 1. Introduction

Wound healing is a complex, systemic, and regulated process to maintain human body functions. In wound healing, the major problem would be wound infections, which impair the healing process. As an act of prevention of this problem, antibiotic medications are prescribed to patients. However, this is ineffective in long-term treatments, especially for chronic wound patients, as this will cause multiresistant bacteria emergence. Therefore, in modern-day wound care, the functionality of wound dressings must be enhanced and addressed to properly manage and care for the wound as it may severely threaten an individual’s quality of life and health.

Cotton fiber is the most recognized natural fiber, which has been widely used in the textile industry. In the biomedical field, cotton fiber has been modified to have antimicrobial properties [1], water repellency [2], and other enhancements of mechanical properties to work efficiently as a wound dressing. Cotton threads have a high specific area, are adjustable in shape, and have high absorption capability, which is good to absorb exudate, reduce blood loss, and keep the wound area free from other debris [3,4]. A minor setback is when a cotton thread wound dressing dries up, it tends to stick to the wound area during the removal process [5]. The removal of the dressing might cause discomfort to patients, causing secondary damage to newly developed tissue, and requires a tedious cleaning process. Therefore, the cotton thread should be modified to have properties that permit balanced and maintaining moisture at the wound site to prevent the dressing from adhering to the wound, reducing pain and discomfort, thus accelerating wound healing.

Carboxymethyl cellulose (CMC) is used in the biomedical and pharmacology fields as an enzyme immobilizer, absorbent, r wound healing, and drug delivery. CMC is biocompatible with other biomaterials. Hence, CMC-based biomaterials with antibacterial properties for wound healing and tissue engineering could be fabricated [6]. CMC-coated wound dressing is more flexible, absorbs exudate, retains moisture to boost angiogenesis and autolytic debridement [7], is nontoxic to humans, and is water soluble [8]. Salt CMC derivatives, such as sodium CMC (Na-CMC) and calcium CMC (Ca-CMC), are commonly used. Na-CMC has an excellent compatibility with human skin, which shows a high film-forming capability and is proven to effectively eliminate microbial growths from wound beds through microbe adhesion and promote wound healing [9]. It is due to Na-CMC substituted carboxylated group (-COO) in the backbone along its cellulose chain. Besides, Na-CMC has been used to fabricate cellulose-based coating through chemical cross-linking as it can form a cross-linked network through ionic bonding, hydrogen bonding, or polymer–polymer interaction [10]. With these advantages, CMC-based biomaterials are widely used in wound dressing applications [7] to prevent wound infections. As coating agent, CMC improve thread quality and mechanical strength [11]. However, CMC on its own has poor mechanical properties. Therefore, citric acid (CA) was used as a crosslinker in this study. CA is present in citrus fruit, such as lemons and oranges. This natural organic acid consists of three carboxylic groups. CA is used in food processing and is now known in the biomedical field for its excellent antimicrobial and antioxidant properties. CA is also beneficial in the pharmaceutical application as it is used in various applications, such as cross-linking, interaction between molecules, and coating agents [12].

Oven drying (OD) is a conventional technique used with a standard temperature–time combination [13]. It is considered convenient since it can accommodate a large number of samples and has relatively rapid and precise temperature control to reach the desired temperature. However, the drawback is that temperature variations might occur due to the samples’ size, weight, and position in the oven, which affect the total removal of moisture from samples, the risk of losing volatile substances or compounds, and sample decomposition during the long drying process [14,15,16]. In addition, sample decomposition is undesirable in wound dressing materials as it may have an immense degradable effect on temperature-sensitive medicinal substances. A more time-saving method, such as an infrared (IR) dryer, is viewed as an alternative to overcome this problem. IR drying provides rapid heating and moisture removal. It serves many advantages, such as high energy efficiency, faster heat transfer rate, and maintaining high-quality products [17,18]. During the drying process, the IR wavelength radiation emitted from the heat source passes through the wet sample and increases the temperature internally without heating the surrounding air [15]. The IR penetration causes water molecules to vibrate, which leads to heating [19]. The heat provided by IR interacts with the samples’ internal structure and facilitates heating from the inner to the outer layer through the radiation and convection thermal phenomena, causing a decrease in moisture content through the evaporation process [19,20]. IR has been shown to provide more advantages in the drying method because it provides uniform heating, has a higher heat transfer rate, reduces processing time and energy, and improves the material quality [15,20]. Table 1 shows a comparison between IR drying and other drying techniques based on the cost-effectiveness, drying time, product quality, and advantages and disadvantages of the drying techniques. Therefore, a distinct comparison between oven and IR drying and a combination of both with regard to the quality of cotton thread wound dressing will be measured.

Our study aims to evaluate the effect of drying methods to CMC cross-linked with CA on cotton thread fibers’ mechanical strength and its wettability properties for wound dressing enhancement. To achieve this aim, we investigate how different coating layers of CMC cross-linked with CA onto cotton thread fibers are influenced by different drying regimes. Our experimental approach evaluated whether drying regimes and two different applied coating layers can potentially affect the cotton thread absorption capability, surface morphology, and mechanical properties. The coated cotton thread moisture content and water absorption were determined. The CMC-coated cotton thread surface was visualized using an optical microscope (OM), and the mechanical properties, such as tensile strength, were assessed. Our results stipulate that a single-layer coat of CMC cross-linked with CA coated cotton thread undergoing IR drying shows better property enhancement of the cotton thread fibers than the oven or oven + IR drying method.

## 2. Materials and Methods

### 2.1. Raw Materials 

Mercerized cotton thread (3-ply with 100% cotton) was purchased from Coats Cotton (Hungary, Europe). Solid powder sodium carboxymethyl cellulose (Na-CMC) (CAT. NO: 419273) with medium viscosity and a degree of substitution of 0.65–0.90 and citric acid (anhydrous, M*w*: 192.12) (CAS. NO: 77-92-9) were purchased from Sigma-Aldrich (St. Louis, MO, USA). 

### 2.2. Preparation of CMC and Citric Acid Coating Solution

About 2% (*w*/*v*) of CMC as a coating material was dissolved in distilled water (dH_2_O) by stirring with a magnetic stirrer, forming a homogeneous aqueous solution. Then 4M CA was dissolved in dH_2_O. The 2M and 3M CA were prepared by diluting 4M CA.

### 2.3. Design of Coating Processes

Cotton thread fibers were washed before coating with 2% detergent solution and 80% ethanol and were oven-dried at 60 °C for 15 min. The coating process was based on previously published results [22] with modifications. The 2% CMC was prepared and allocated in 25 mL syringes with a 20-gauge needle size. Cotton thread fibers were soaked in the syringes with CMC solution for 18 h.

After the threads were soaked, the threads were pulled out from the syringe and were immediately submerged into beakers containing heated 2M, 3M, and 4M CA. The threads were left immersed for 1 h at 90 °C with constant stirring to promote CA and CMC interaction [23,24]. After the immersion, the excess CA was removed using filtered paper, and the thread groups were separately dried using OD and IR drying techniques at 65 °C for 30 min. In addition to that, OD drying was conducted in a convection oven (Memmert UFTS, Germany). For IR drying, the distance between the IR lamp and the sample in the drying machine was fixed at 7 cm (Desktop Infrared Drying Machine (GW-200H), Hoystar^®^, Guandong, China). During the sequential drying regime (OIR), drying was first conducted in the oven at 65 °C for 15 min, followed by IR drying for another 15 min, similarly at 65 °C, making a total drying time of 30 min. 

The coating process was altered in six samples, which were divided into three sets. The first three sample groups were set as control, where no coating of CMC and CA was be performed. The second set consisted of the sample groups where one layer of CMC was performed. The third set is where two CMC layers were applied under the same conditions, with respective drying regimes after each coat. Once all samples were dried, the dry coat weight was measured as a control sample by weighing. Table 2 lists the coating parameters used to design the coating process. 

### 2.4. Determination of Physical Characteristic of Uncoated and Coated Cotton Threads

Basic physical properties, such as basis weight, thickness, and moisture content, were assessed using uncoated and CMC-coated samples. The basis weight of uncoated and coated samples was determined using an analytical balance (A&D Compact Analytical Balance, HR-250AZ), and the sample thickness was measured with an ABS Digital Thickness Gauge (Code: 547-301) (Mitutoyo, Kanagawa, Japan). After samples were dried according to the regimes, samples’ moisture content was evaluated using a moisture analyzer machine (A&D Moisture Analyzer, MX-50). 

### 2.5. Evaluation of Mechanical Properties

Tensile strength (TS), modulus, and percent elongation at break were measured using the Servo Control System Desktop Tensile Strength Tester AI-3000N (Qingdao, China). The test was performed based on the ASTM D3822 standard (Standard Test Method for Tensile Properties of Single Textile Fibers). Before running the tensile test, all samples were conditioned overnight in a drying cabinet at room temperature and 59.4% relative humidity (Weifo, Taiwan). The distance between the two clamps was set to 50 mm, and the strain rate speed was constant for all samples at 10 mm/min with a 10 kg load cell. The samples were cut at a length of 70 mm. Both coated and uncoated thread samples were secured with masking tape at both ends and inserted between the clamps to avoid fiber slipping during testing. The average fibers’ strength and percent elongation were calculated based on five measurements. The tensile strength was obtained by dividing the average tensile load and average fiber diameter. 

### 2.6. Determination of Water Absorption and Moisture Content

#### 2.6.1. Water Absorption

Water absorption was evaluated by immersing samples into distilled water. Each thread sample was immersed for 0.5, 1, 3, 5, 7, and 30 min. The samples were weighed before and after immersion, and then the water absorption was calculated using Equation (1).

(1) $$W\;(\%)\;=\;\left[\frac{m_{a}}{m_{o}}\right]\;\times \;100$$

where W is the water absorption of the samples in (%), *m_a_* is the sample mass (mg) after water absorption, and *m_o_* is the initial mass of the samples before water absorption [25].

#### 2.6.2. Moisture Content

Moisture content was determined by recording the sample mass at similar time intervals, and moisture content was calculated as shown in Equation (2).

(2) $$\mathrm{MC}\;(\%)=\frac{Mcws-Mcs}{Mcs-Mc}\;\times \;100$$

where *Mcws* is the container plus wet sample mass (mg), *Mcs* is the mass of the container plus dried sample (mg), and *Mc* is the container mass (mg) [26].

### 2.7. Surface Analysis

#### 2.7.1. Surface Morphology

The surface topography of coated threads was observed under a Dino-Lite Optical Microscope and analyzed with the DinoCapture V2 program. The samples were observed under 440× magnification.

#### 2.7.2. Attenuated Total Reflection–Fourier-Transform Infrared Spectroscopy (ATR–FTIR)

FTIR was carried out using Alpha Platinum-ATR (Bruker, Massachusetts, United States). FTIR spectra of the samples were recorded at 32 scans with a resolution of 4 cm^−1^ and a wavenumber range between 400 and 4000 cm^−1^. 

### 2.8. Antibacterial Analysis

Fabricated cotton threads with different concentrations of CA and different coating layers were investigated based on a previous published study [27], which was conducted using agar plate diffusion method (GB/T 20944.1-2007). Gram-negative (*Escherichia coli*) and Gram-positive (*Staphylococcus aureus*) bacteria were diluted into a suspension (1 × 10^6^ CFU/mL) and spread on the sterile agar plate. The fabricated cotton threads were placed transversely onto the agar surface to ensure contact, and the plates were incubated at 37 °C for 24 h. The zone of inhibition was evaluated by the inhibition zone diameter values of the samples and calculated based on the formula shown in Equation (3).

(3) $$H\;(\mathrm{mm})=\frac{D-d}{d}$$

where H refers to the zone of inhibition (mm), *D* refers to the total diameter of the sample and inhibition zone (mm), and *d* refers to the diameter of the cotton thread (mm).

### 2.9. Statistical Analysis

The selected data were given as mean values with standard deviations. The number of replicates was constant, where *n* = 3 replicates for each observation. Analysis of the data obtained from the experiments was performed using the ANOVA function in Microsoft Excel with a confidence level of *p* < 0.05.

## 3. Results and Discussion

### 3.1. ATR–FTIR Characterization of Uncoated CT and CT Coated with CMC Cross-Linked with CA (CT/CMC + CA)

**Figure 1:**
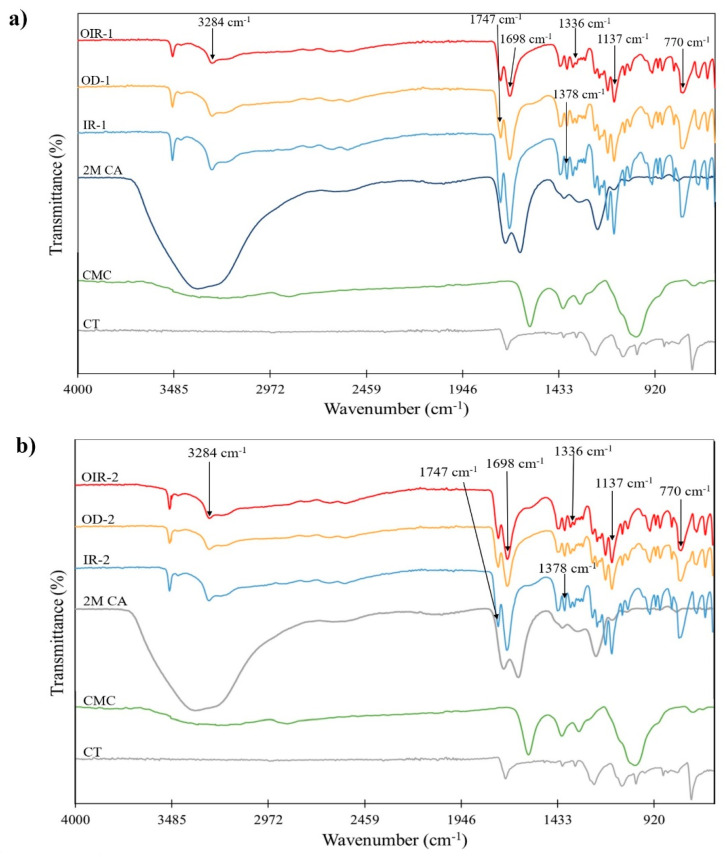
FTIR spectra of (**a**) single-coated and (**b**) double-coated CMC cross-linked with 2M CA in different drying regimes.

The presence of CMC and CMC + CA coating on CT was confirmed by FTIR analysis. The ATR–FTIR measured in this study is within the mid-IR spectrum of 400–4000 cm^−1^. Figure 1 shows the spectrum of the pristine CT and CT coated with CMC cross-linked with 2M CA samples at different coating layers with different drying regimes. CA spectra showed characteristic peaks at 3290 cm^−1^ corresponding to O–H stretching for H_2_O and 1745 and 1698 cm^−1^, which matched with C=O stretching of carboxylic acid [28]. As regards CMC, FTIR peaks were observed at 3356, 1587, and 1051 cm^−1^ corresponding to O–H stretching, carboxylate C=O stretching, and C–O–C stretching, respectively [29]. Based on Figure 1, there is no significant difference between coating layers at *p* > 0.05, which proves that coating layers have no adverse effect on the chemical functionalities of the samples. Additionally, similarities among the spectrum prove that CA was homogeneously cross-linked with CMC. However, as regards different drying regimes, there are slightly different transmittance intensities to the CMC + CA spectra. 

**Figure 2:**
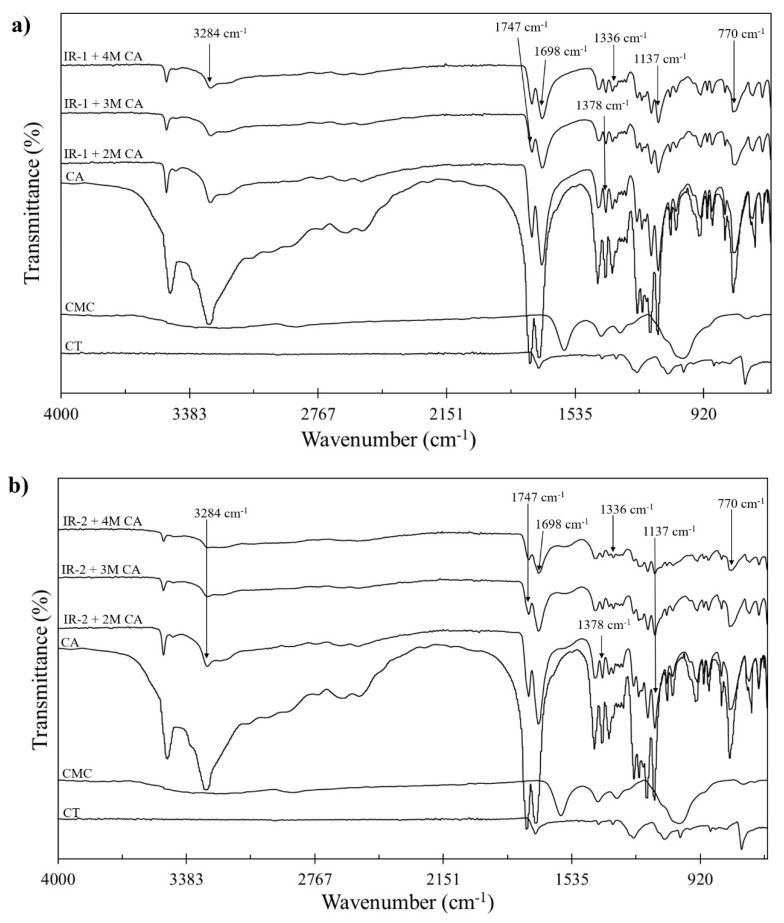
FTIR spectrum comparison between (**a**) single-coated CMC cross-linked with 2M–4M CA, (**b**) double-coated CMC cross-linked with 2M–4M CA.

After different concentrations of CA were added to the CT/CMC samples, peaks can be observed at 3284, 1747, and 1698 cm^−1^, and the cross-linking mechanism involves the attachment of carboxylate groups of CA to the hydroxyl group of CMC, which could be attributed to the esterification between citric acid and CMC, demonstrating chemical linkage formation among them [30]. In addition, the peaks at 1747 and 1698 cm^−1^ became more intense, with the increase in CA concentrations, as shown in Figure 2a,b, stipulating a higher cross-linking reaction. The ATR–FTIR results suggested that there was an occurrence of cross-linking interaction between CT/CMC and CA. Figure 2a,b demonstrates a similar observation at 3284 cm^−1^, where peaks at lower concentration showed less intense peaks due to the esterification reaction during cross-linking [31]. Thus, the results obtained are in tune with those of previous studies [28,32].

The slight differences in transmittance intensity among different drying regimes are due to the transmittance value among the drying regimes. IR has the highest transmittance values, followed by OD and OIR drying regimes. A high transmittance value indicates a large population of specific functional groups that emits vibrational energies, which corresponds to the reflected light [33]. The large population of specific bonds suggests that IR is an effective drying regime since CMC + CA is more concentrated due to due to the efficient moisture removal. As the moisture is efficiently removed from the coated CT, macromolecular compaction occurs, and the population of specific bonds per mm^2^ increases. The concentrated amount of CMC + CA coating in IR-dried samples causes them to emit a high transmission value to a specific functional group population bond. More apparent differences are shown in Figure 1 of OD and OIR samples, regardless of layers. Even though the amounts of CMC and CA used were controlled, distinct transmittance values among different drying regimes indicated different efficacies of moisture removal. The efficiency of moisture removal will be highlighted in Section 3.2.

**Figure 3:**
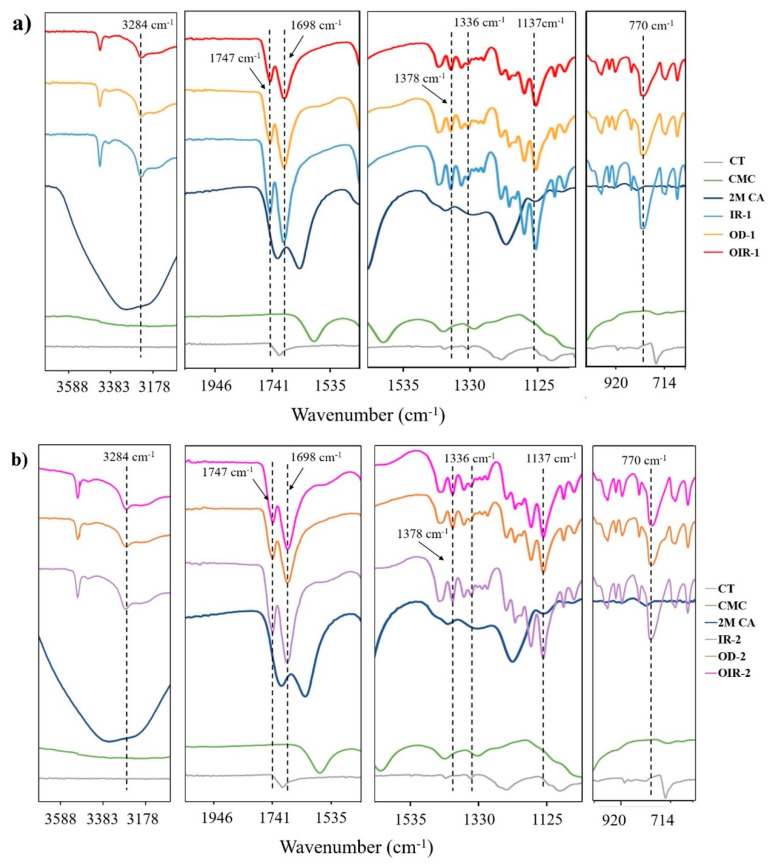
Enlargement of the FTIR spectra of (**a**) single-coated and (**b**) double-coated CMC cross-linked with 2M CA in different drying regimes.

In Figure 3, four enlargements of Figure 1, which exhibit the FTIR spectra of dried samples by different drying regimes on a specific range of wavelengths, are shown. The stretching bands of the functional groups of the IR-dried samples were similar to those of the OD and OIR samples. According to Figure 2, the OH group appeared within the broad adsorption peak in all drying methods. A slight decrease in the wavenumber was observed by comparing the CT/CMC + CA samples and uncoated CT samples. The carbonyl peaks of CMC + CA emerged at 1747 and 1698 cm^−1^ in the spectral region between 1750 and 1600 cm^−1^ for all samples, both in single and double coats. There was a shift towards a smaller wavenumber within this wavenumber, and a similar trend was observed at peaks of 1378 and 1137 cm^−1^. When the peaks shift to a smaller wavenumber, this indicates that the molecule within this wavenumber has increased in its mass. The vibration frequency is inversely proportional to the mass of the vibrating molecule. Therefore, the heavier the molecule, the lower the vibration frequency, thus the smaller the wavenumbers [34]. At a wavenumber of 770 cm^−1^, no changes were observed between the drying regimes. This result helps confirm that different drying regimes did not affect the sample compositions.

### 3.2. Physical Properties of Uncoated CT and Coated CT/CMC + CA

#### 3.2.1. Basis Weight and Thickness 

As seen in Table 3, the basis weight and thickness of coated CT are directly proportional to the coating layer and concentration of solids in the coating formulation. Coated samples gained the basis weight and thickness as expected compared with uncoated samples, with double-coated samples accumulating the highest weight gain and thickness. CT coated with CMC increased its weight and thickness but not as much as CT coated with CMC + CA. The substantial increase in CMC + CA samples’ weight proved that cross-linked CMC + CA has better coating efficiency due to the accumulation of CA attached to the -OH group of CMC after the esterification process. It was also found that the CT/CMC weight and average thickness increased significantly with CA concentration. This is because the higher the CA concentration, the higher the probability of CA to cross-link with CMC. At a higher cross-linking degree, CMC + CA becomes more viscous, allowing more intermolecular bonds to form between CMC and CA [35], thus increasing their weight and average thickness. For medical purposes, such as skin grafting, it is proposed that the dressing film should be thinner than 400 µm to allow vascularization for nutrient delivery to the epidermis [36]. Therefore, the coating thickness of the samples was fabricated within the desirable range except for the samples OIR-1 and OIR-2 at 4M CA.

For the curing process via several drying regimes, IR samples showed the smallest thickness, followed by OD and OIR samples in single- and double-coated CT. A higher sample thickness on OIR samples is due to the unequal heating energy imposed in the transitioning process between the samples from OD and IR in the sequential drying regimes of OIR. Meanwhile, samples dried via IR and OD provided more uniform heating, allowing moisture from the samples to be removed efficiently and causing a substantial difference reduction in sample size [15]. Nevertheless, based on sample thickness, IR is the most effective, followed by OD and, finally, OIR. OIR is not as effective as expected, particularly as a solitary drying regime. It is due to the uneven energy used to dry the samples, and the samples are prone to absorb moisture when transitioning from one drying regime to another. Therefore, the ineffectiveness of OIR will have to prolong the drying time or increase the heating energy for the sample to be dried effectively.

#### 3.2.2. Moisture Content

Moisture content was determined to evaluate the amount of moisture in the conditioned uncoated and coated CT. After the drying process, the extent of destruction to the lamellar structure of the macromolecule network will differ among different drying regimes. Hence, the ability to absorb the surrounding moisture will determine the severity of drying regimes to the CMC-CA polymer networks. The ability to retain moisture is crucial for the CMC + CA coating on CT to function as a functional wound dressing. According to the Gibbs–Donnan effect theory, in regard to the transportation across cell membranes, if the Gibbs–Donnan equilibrium between the exterior surroundings and the wound bed is achieved, the increase in intracellular ions would cause cells to swell due to the osmotic influx of water. Low ionic strength can lead to a high distribution of ion mobility, establishing the Gibbs–Donnan equilibrium between the external environment and wound bed, and thus providing a stable structure for the coated CT to retain moisture and/or for drug delivery systems for wound healing [37].

Based on Table 4, the moisture content of CT/CMC + CA gradually decreases with the increase in the coating layer in all drying regimes. Increases in coating layers resulted in an increase in ionic strength, which reduced the number of mobile ions within the CMC + CA coating matrix, thus reducing the CMC + CA coating structure and limiting the moisture content of CT/CMC + CA [38]. The result is similar to the findings of other biomaterials, such as membrane [39] and hydrogel films [29,40], that are cross-linked with CA. Meanwhile, IR-1 shows the highest moisture content among the single-coated samples, followed by OD-1 and OIR-1. The same trend can be observed for the double-coated samples. The increase in moisture content at a low concentration of 2M CA in all drying regimes shows that the CMC + CA coated on CT can retain moisture. This is caused by the hygroscopic nature of CT/CMC + CA with hydroxyl and polar groups available and the interfacial area between CT and the CMC + CA matrix [41]. On the other hand, at a higher CA concentration (>2M) and double-coated CT/CMC + CA samples, a low moisture content was obtained, and it correlates with the cross-linking effect, which reduces the number of free hydrophilic groups in the coating, thus limiting molecular mobility, and binds the polymer together tightly [40].

The findings are consistent with the basis weight of coated samples portrayed in Table 2. A higher moisture content by IR showed that IR is more effective in removing the moisture than OD and OIR. At a similar temperature and time, unlike OD, IR minimized the capillary force driven by the surface tension of water, hindering the destruction of its lamellar structure. For OIR, the lowest moisture content absorbed by them is due to the sequential drying regime unable to effectively remove moisture during the drying process, resulting in high capillary force and the destruction of OIR samples’ lamellar structure, rendering them unable to retain moisture. 

### 3.3. Surface Morphology of Uncoated CT and Coated CT/CMC + CA

**Figure 4:**
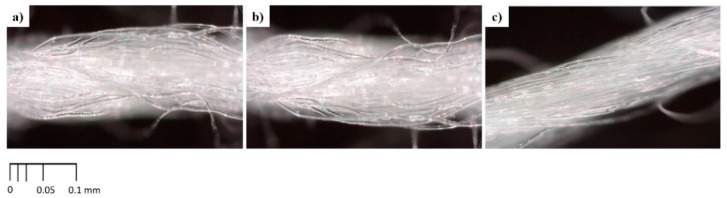
Uncoated cotton thread surface of (**a**) CT-A, (**b**) CT-B, and (**c**) CT-C.

**Figure 5:**
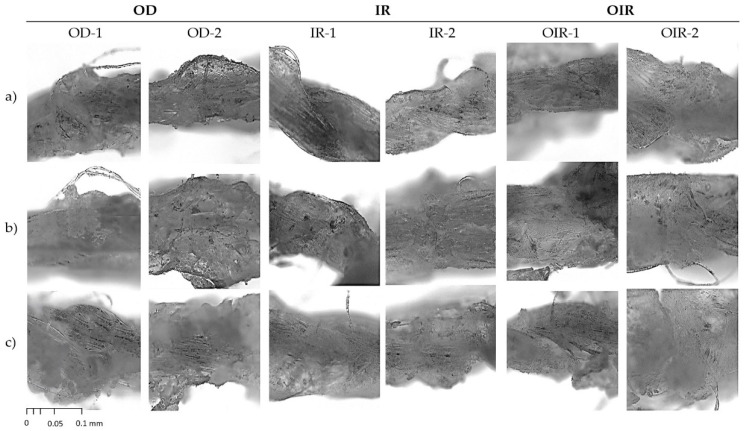
Observation under an optical microscope of a single coat (OD-1, IR-1, and OIR-1) and double coat (OD-2, IR-2, and OIR-2) cross-linked with (**a**) 2M CA, (**b**) 3M CA, and (**c**) 4M CA under different drying regimes with a scale at 0.1 mm.

The surface morphology of uncoated and coated samples is shown in Figure 4 and Figure 5, respectively. In Figure 4, the uncoated samples (CT-A, CT-B, and CT-C) showed no significant difference in terms of morphology and embodied plenty of empty spaces among the cotton fibers, making them porous. This will allow CMC + CA solutions to penetrate easily during the coating process.

Figure 5 shows CT morphology after coating (single and double coat) and the respective drying process. In comparison with Figure 4, Figure 5 shows that the empty spaces between the cotton thread fibers of single- and double-coated samples are filled with coated materials. In Figure 5, all samples show that the CMC-CA were homogeneously and evenly coated on the CT surfaces. The cross-linking between CT/CMC + CA involves the interaction of covalent esters composited as a coating layer that enhances the physicochemical properties of the CT. This is a crucial feature for CT/CMC + CA to achieve a stable structure. The stable structure permits CT/CMC + CA moisture retention enhancement, as shown in Section 3.2.2, and further improvement of CT/CMC + CA mechanical properties and absorption capability. Therefore, CT/CMC + CA can be utilized as a functional wound dressing.

As shown in Figure 5, the cotton threads coated with CMC cross-linked with CA presented textured surfaces, particularly when a high concentration of CA (4M CA) was used, which affected their physical properties. CA-cross-linked CT/CMC thread had more protrusions than uncoated cotton threads, especially at a high CA concentration, indicating increased surface roughness. On the other hand, double-coated samples (OD-2, IR-2, and OIR-2) showed excessive citric acid crystal formations due to the high CA amount present in the coating, promoting a coarser surface on cotton thread. The enhancement of surface roughness in a medical application provides a better interaction zone between the dressing with the cells and tissue surface, allowing dermal fibroblast adhesion [42], which enhances cell adhesion and cell proliferation [43]. This aids in strong protein adhesion to the wound dressing surfaces, implying that a rough surface can stimulate cell signaling by imitating similar cues as the extracellular matrix network [44,45,46].

IR-1 and IR-2 samples visually showed a smoother coating topography than OD and OIR drying samples, which exhibited rougher surface coverage. This was due to IR drying involving a more uniform heating mechanism where the temperature of the samples was increased internally [15]. When the samples were heated internally via the IR drying process, disturbance to the polymer interactions scarcely occurred, facilitating a uniform solidification of coating materials interpreted by a smoother topography as depicted in Figure 5. Meanwhile, for OD and OIR samples in Figure 5, nonuniform topography is triggered by the sample’s placement during the drying process. Samples’ positioning promotes temperature variations in the oven environment, which subsequently cause propagation of polymer interactions during the solidification process of coating materials [15,16]. Besides, a sequential drying process of OIR is inefficient due to the change of heating energy between the two drying regimes. In addition, the surrounding factor during the transition of OD to IR drying is exacerbated by moisture absorption and the sudden temperature drop and, thereupon, disturbs the solidification process of coating materials. In essence, due to the uneven heating energy, OD- and OIR-dried samples exhibit a heterogeneous structure and texture. With this in mind, different drying regimes gives different morphology depending on the drying mechanism efficiency [47].

### 3.4. Mechanical Properties of Uncoated CT and Coated CT/CMC + CA

**Figure 6:**
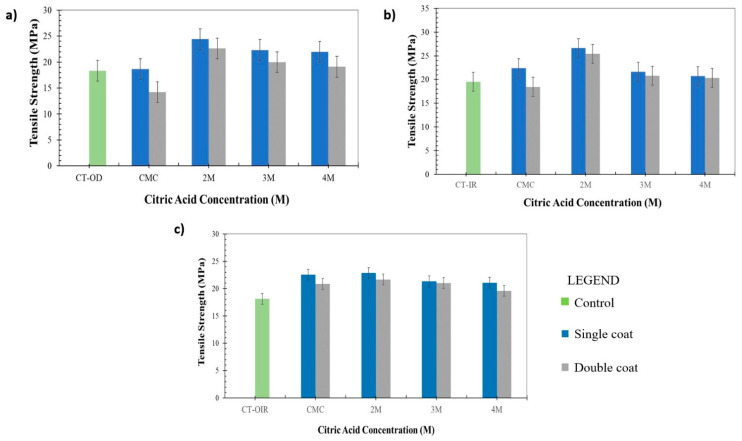
Tensile strength of CT/CMC cross-linked with different CA concentrations dried by (**a**) OD, (**b**) IR, and (**c**) OIR.

**Figure 7:**
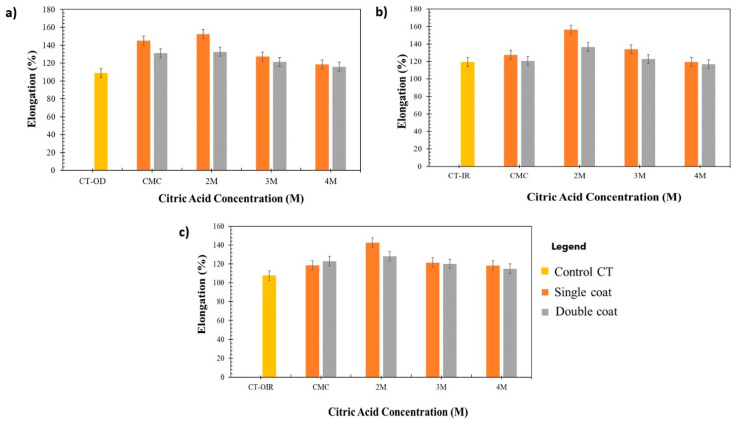
Elongation percentage of CT/CMC samples cross-linked with different CA concentrations dried by (**a**) OD, (**b**) IR, and (**c**) OIR.

The tensile test was performed to investigate the effects of coating layers and drying regimes on the mechanical performances of CT/CMC + CA. The samples’ tensile strength and elongation percentage (%) are presented in Figure 6 and Figure 7.

Figure 6 and Figure 7 show that the tensile strength and elongation percentage of CT/CMC + CA are higher than those of uncoated samples. It is well understood that as tensile strength increases, the elongation percentage decreases and vice versa [48]. However, CA, as the crosslinker in the coating system, increases tensile strength and elongation due to a higher potential of interaction between material and matrix polymer, resulting in increased of intra- and intermolecular cross-linking between polymers [49]. The CT fiber twisting orientation also plays a role in the increase in tensile strength and elongations percentage [50] as the twisting provides extra length, allowing CT to stretch more when a load is applied. The extension increased CT lateral pressure and compaction and, as a result, CT became denser and more coherent to strain. CT has a slightly loose twisting orientation that allows CMC + CA to penetrate and coat individual fiber surfaces, which further improves CT load bearing capacity by intercepting higher loads, thus leading to the improvement of elongation and avoiding early breakage [51]. 

The interactions contributed to a more coherent structure, improving the tensile strength and elongation percentage of CT/CMC + CA. When comparing single- and double-coated samples, single-coated CT had higher tensile strength than double-coated samples. Generally, the tensile strength of composites increases with composite content until a maximum or optimum value is achieved. Once the maximum value is reached, the tensile strength will decrease. A higher tensile strength for single-coated CT for all drying regimes is due to the penetration of the coating solution between the fibers. CMC + CA acts as a space filler, which also contributes to the load distribution, increasing the tensile strength and elongation percentage [52]. However, the reversed scenario was observed for double-coated CT/CMC + CA samples. This is due to the high amount of CMC + CA content in the double-coating layer that reduces the matrix mobility, making it stiffer, hence indicating that the amount of CMC + CA in double-coated CT might have exceeded the optimum composite content of CT/CMC + CA strength [53].

The presence of CA caused significant differences (*p* < 0.05) in the tensile strength. In all drying regimes, regardless of coating layers, the tensile strength and elongation percentage are the highest at 2M CA and continuously decrease afterward. At higher concentrations of CA, namely, 3M and 4M, clumping is prone to occur due to the quick cross-linking process between polymers. When cross-linking occurs at a higher rate, it causes a higher number of CA that might have cross-linked with CMC in the matrix, causing CMC + CA agglomeration. With that being said, a higher concentration of CA leads to clumping [49,54], thus acting as stress concentrators, causing CT to be stiff and resulting in lower mechanical performances [55]. Based on the results obtained, the mixture performed best at a lower concentration of CA (2M) to improve the tensile properties of CT because of the intramolecular and intermolecular interaction forces, which enhanced the coated CT strength due to the formation of a stronger network force in the matrices. In addition, at higher CA concentrations (>2M) and coating layers, high CA content results in the formation of CMC + CA crystals (see Figure 6). This causes the CT/CMC + CA structure to be brittle [56]. Brittleness degrades the mechanical performances of CT by disrupting the elasticity and intermolecular force network in the CMC + CA matrices. This explains the low tensile properties of the above-stated determinant. CA concentrations of more than 2M were shown to be unsatisfactory when conjoined with the double coating process.

Apart from the effect of the coating layer, an apparent distinction between different drying regimes can also be seen in Figure 6. Overall, IR-dried single- and double-coated CTs showed the highest increment in tensile strength and elongation percentage, followed by OD and OIR. The tensile strength of single-coat samples OD and IR improved by 33% and 36%, respectively. In contrast, for double-coated samples, the tensile strength increased by 24% and 30%, respectively, compared with the uncoated samples. As regards OIR, it showed the lowest tensile strength improvement at 26% for single coat and 18% for double coat. The tensile strength between the single- and double-coated samples had a significant difference of *p* < 0.05 through all drying regimes. The significant differences were attributed to a uniform heating pattern of OD and IR drying in the single-coated samples, which allowed moisture to evaporate efficiently [15] compared with the double-coated samples. However, for OIR, despite the significant differences, the regime did not effectively remove moisture from the coated samples, both single and double, as shown in Table 4, under the dry moisture content percentage. A rapid removal of water induced by IR is more focused than the OIR technique, where the differences in heating energy during OIR transitioning affect samples’ tensile properties. In OIR, environmental factors, such as moisture absorption that ensued with a temperature drop during the samples’ transitioning process, should also be considered. Due to this matter, incomplete drying can cause the formation of a void of a channel structure in the coating matrix, which also leads to the presence of moisture during OIR sample fabrication, which disrupts the solidification of CMC + CA due to air entrapment in the coating structure [57]. A heated CT via OD prior samples transferred to IR creates osmotic differences between samples and the environment. Thus, moisture will be absorbed by the CT, which leads to a temperature drop. Consequently, OIR CT/CMC + CA samples have irregularities in their chemical and physical interactions due to the fluctuating sample temperature, which were later signified by the OIR sample morphological structure and tensile strength.

### 3.5. Effects of Drying and Coating Layers on Water Absorption

**Figure 8:**
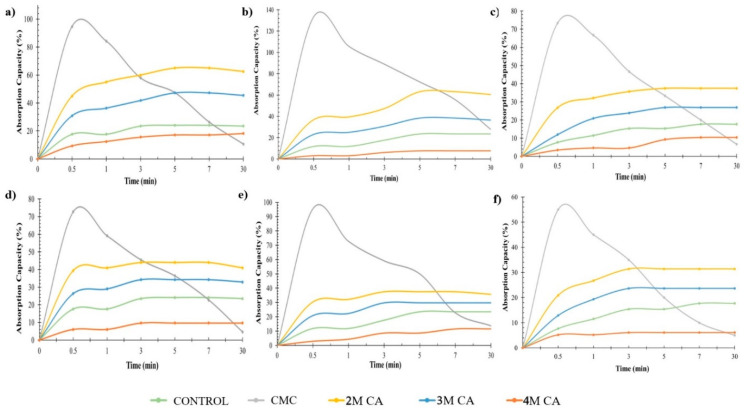
Water absorption behavior of CT/CMC + CA based on different drying methods. (**a**) OD-1, (**b**) IR-1, (**c**) OIR-1, (**d**) OD-2, (**e**) IR-2, and (**f**) OIR-2.

The average water absorption of CT/CMC + CA samples versus time is plotted in Figure 8. It can be observed that the water absorption of CT/CMC + CA samples increased with an increasing time and equilibrium after 5 min of immersion for single-coated samples and 3 min of immersion for double-coated samples in all drying regimes. Due to the relatively higher CMC + CA content in double-coated samples than single-coated samples, their water absorption capability is reduced, causing it to reach its maximum capacity faster than single-coated samples. When CMC + CA double-coats the CT, the solid content increases due to the cross-linking interactions, creating more networks between CMC and CA, thus reducing the mobility of the ionized ionic group in the CMC + CA matrix due to a stiffer and denser structure [31,56]. On the other hand, in a single CMC + CA coating, the carboxyl groups (ionic group) in CMC are known to be highly hydrophilic. When ionized (COO-), it increases the electrostatic repulsion between intramolecular and intermolecular interaction forces to open the polymer matrix, increasing the water absorption properties to the materials [58].

According to Figure 8, an obvious pattern is seen between different concentrations of CA. A higher CA causes a continual reduction in water absorption, with the most conspicuous at 4M CA. The CT coated with CMC + CA at 4M CA water absorption is even lower than the CT itself. Even though CMC and CA are naturally hydrophilic, excessive cross-linking events between polymers can induce the hydrophobic character. The induction is triggered by physical changes, where the cross-linking effect of CA results in the coat being hydrophobic with a high-density structure [28]. A high CA concentration can increase the solid content of the CT/CMC + CA matrix, which causes the cross-linking interactions to create a stronger bond between CMC and CA, resulting in a compact structure and higher density [34,58]. Besides, the carboxyl groups (ionic group) in CMC are known to be highly hydrophilic. When ionized (COO-), it increases the electrostatic repulsion between intramolecular and intermolecular interaction forces to open the polymer matrix, increasing the water absorption properties to the materials [58]. As proven in the surface morphology analysis, the different drying methods affect the reinforcement of CMC + CA into the CT structure. For this reason, water absorption is also altered, and the hydrophilicity of CMC + CA is influenced by different drying methods. However, other factors, such as coating layers, contribute to the result and must be considered. In this study, a single layer of CMC + CA was sufficient to coat the CT and penetrate the CT fiber. However, by adding double layers, the coating structure became stiffer and denser. This resulted in moderate changes in the CT/CMC + CA properties, such as tensile strength, moisture absorption, and water absorption, when a single layer was applied, and a significant impact when double layers were added, regardless of the drying regimes used.

### 3.6. Antibacterial Activity of Coated CT

From a medical perspective, *Escherichia coli* (*E. coli*) and *Staphylococcus aureus* (*S. aureus*) are the most common representatives for Gram-negative and Gram-positive bacteria, respectively, that cause infections in the human body. Gram-negative bacteria (*E.coli*) tend to be more resistant to antimicrobial agents that Gram-positive bacteria (*S. aureus*) because of the presence of the additional protection afforded by the outer membrane (peptidoglycan layer). However, in this study, *E. coli* growth was inhibited due to the presence of CA, which weakened the outer membrane of Gram-negative bacteria [59]. The result is supported by that of a previous study, where CA exhibited more inhibition to *E. coli* compared with *S. aureus* [60]. *S. aureus* was more resistant to the acidic environment since *S. aureus* is better at tolerating and adaptive to stress [61]. 

**Figure 9:**
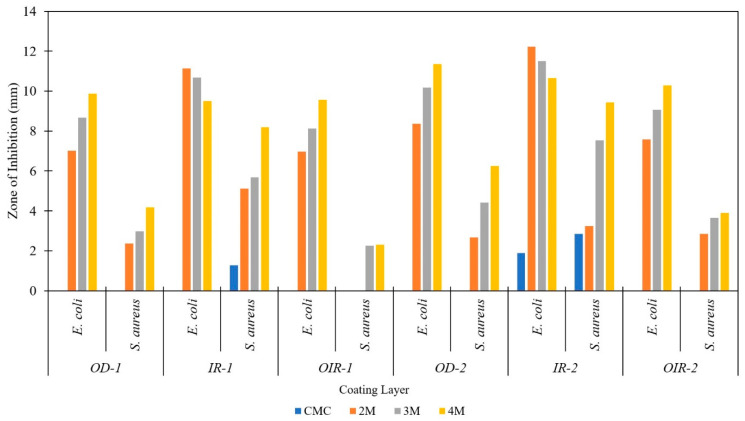
Antibacterial activity of fabricated cotton thread with different coating layers of CT/CMC cross-linked with different CA concentrations.

As shown in Figure 9, the zone of inhibition increases with the increase in coating layer and CA concentration for OD and OIR, which is attributed to the increase in reactive oxygen species (ROS). ROS is responsible for the inhibition and causes interruption of the bacterial cell wall synthesis process, growth biosynthesis inhibition, DNA transcription process interference, and metabolic pathway chain reaction disruption occurring in the bacteria cell [62]. However, IR samples showed lower CA concentrations able to inhibit *E.coli* growth. At 2M CA, both IR-1 and IR-2 samples showed the highest inhibition for *E.coli*. It is due to CA, which acts as a permeabilization agent able to inhibit *E. coli* growth by causing cell aggregation [59] and bacterial toxicity by blocking the permeability of the outer membrane [63,64]. Both bacterial species are resistant to CMC except for the IR-2 samples, while the IR-1 CMC sample only showed inhibition to *S. aureus* bacteria. The effectiveness of IR drying might also be the reason why a lower CA concentration is sufficient to inhibit bacterial growths. This finding might be due to the effective drying of CMC through IR drying, which permits its antimicrobial property to be preserved. It is achieved due to the uniform heating provided by the IR drying technique, as mentioned in Section 3.3. These findings indicate that CMC with CA as a cross-linker coating dried by IR improved the antibacterial property of cotton threads.

## 4. Conclusions

Based on these findings, the mechanical properties of coated samples are significantly affected by the drying method used at *p* < 0.05. However, the IR drying method is the best-method approach. A single coat of CT/CMC + CA showed the best properties among all samples, with single-coated samples dried via IR drying, exhibiting a less brittle and less dense structure with a slightly smoother surface as well as enhanced water absorption and tensile strength in contrast to double-coated samples. The ATR–FTIR analysis spectra corresponding to C=O from COOH of CA indicated that cross-linking between CMC and CA interaction occurred between CT/CMC-coated samples. Single-coated CMC cross-linked with 2M CA cotton thread dried with IR proved to be a successful candidate for the fabrication of cotton thread with beneficial properties for wound dressings and biomedical applications.

## Figures and Tables

**Table 1:** Comparison between drying techniques.

Drying Techniques	Cost-Effective	Drying Time	Product Quality	Mechanism	Advantages	Disadvantages	Ref.
Oven/ convective	Low	Long. Depends on hot air temperature and air velocity	Depends on the parameter of drying	Moisture exchange between sample and the hot air flow through drying chamber	Long shelf life, simple and easy operation	Exposure to oxidation, crust formation of sample surface due to high temperature	[20,21]
Infrared	Low	Short. Increases with air velocity and decreases with IR intensity	High	Sample is exposed to electromagnetic radiation where heat is transferred from heat source to product surface	Environmentally friendly, provides heat homogeneity, short heating time and low energy consumption, has high accuracy, and increases manufacturing efficiency	Exposure to high heat leads to burns; the penetration depth is limited. Prolonged exposure causes tissues rupture. Infrared is not sensitive to reflective properties.	[18,20,22]
Microwave	Low	Reduces drying time	High	Volumetric heating occurs when electromagnetic waves pass through sample, leading to molecule oscillation that generates thermal energy to remove water	Volumetric heating spread through sample reduces drying time	Leads to damaged product because of improper heat control and mass transfer	[21]
Freezing	High	Slow	Low	Two steps: (1) Freezing water molecules in sample (2) Frozen solid samples are heated to induce moisture sublimation	Prevents oxidation, minimal shrinkage and soluble solid shift, volatile compound retention, maintaining porous structure	High facility cost. Could cause major loss of aromatic components	[22,23,24]

**Table 2:** Design of coating trial with a defined number of layers and drying regimes. Trials with one layer of CMC are indicated by OD-1, IR-1, and OIR-1, while trials with two CMC layers are indicated by OD-2, IR-2, and OIR-2. CT-A, CT-B, and CT-C are the uncoated samples.

Samples	Coating Layer	Drying Method
CT-A	0	Oven
CT-B		IR
CT-C		Oven + IR
OD-1	1	Oven
IR-1		IR
OIR-1		Oven + IR
OD-2	2	Oven
IR-2		IR
OIR-2		Oven + IR

**Table 3:** Physical measurement of uncoated and coated cotton thread samples.

Samples	Coating Layers	CA Concentration (M)	Drying Regime	Basis Weight (UCS) (mg)	Basis Weight (CS) (mg)	Average Thickness (µm)
CT-A	0	n.a.	Oven	3.4 ± 0.2	-	112.5 ± 1.1
CT-B		n.a.	IR	3.3 ± 0.2	-	112.8 ± 1.0
CT-C		n.a.	Oven + IR	3.2 ± 0.3	-	106.5 ± 1.3
OD-1	1	CMC	Oven	3.2 ± 0.2	3.5 ± 0.1	127.8 ± 1.1
		2		3.3 ± 0.1	4.7 ± 0.3	183.5 ± 1.2
		3		3.2 ± 0.2	5.1 ± 0.3	249.0 ± 1.0
		4		3.4 ± 0.1	6.0 ± 0.2	277.0 ± 1.1
IR-1	1	CMC	IR	3.1 ± 0.2	3.6 ± 0.3	125.3 ± 1.2
		2		3.2 ± 0.1	4.9 ± 0.6	178.5 ± 1.0
		3		3.0 ± 0.3	5.9 ± 0.6	182.3 ± 1.0
		4		3.3 ± 0.1	6.7 ± 0.4	226.0 ± 1.1
OIR-1	1	CMC	Oven + IR	3.3 ± 0.1	3.5 ± 0.3	247.8 ± 1.2
		2		3.0 ± 0.2	3.9 ± 0.3	336.0 ± 1.2
		3		3.2 ± 0.1	4.3 ± 0.1	362.5 ± 1.3
		4		3.0 ± 0.3	4.8 ± 0.4	444.8 ± 1.3
OD-2	2	CMC	Oven	3.4 ± 0.1	6.3 ± 0.2	136.5 ± 1.1
		2		3.4 ± 0.1	10.0 ± 0.6	256.1 ± 1.3
		3		3.5 ± 0.1	11.7 ± 0.1	267.8 ± 1.4
		4		3.4 ± 0.1	14.4 ± 0.1	309.0 ± 1.8
IR-2	2	CMC	IR	3.2 ± 0.1	6.5 ± 0.2	129.5 ± 1.3
		2		3.1 ± 0.2	10.8 ± 0.1	241.7 ± 1.5
		3		3.3 ± 0.1	12.1 ± 0.2	283.8 ± 1.1
		4		3.3 ± 0.1	18.1 ± 0.2	286.8 ± 1.1
OIR-2	2	CMC	Oven + IR	3.1 ± 0.1	6.4 ± 0.2	285.8 ± 1.3
		2		3.4 ± 0.2	9.3 ± 0.1	374.0 ± 1.5
		3		3.0 ± 0.1	10.0 ± 0.1	381.0 ± 1.1
		4		3.5 ± 0.2	14.4 ± 0.2	446.5 ± 1.3

**Table 4:** Moisture content (%) of CMC cross-linked with different concentrations of CA-coated cotton thread samples.

Samples	Coating Layer	(CA) (M)	Drying Regime	Moisture Content (%)	
				Dry	Wet
CT-A	0	n.a	Oven	3.08 ± 0.04	92.3 ± 0.02
CT-B		n.a	IR	3.05 ± 0.03	100 ± 0.02
CT-C		n.a	Oven + IR	3.11 ± 0.03	84.6 ± 0.03
OD-1	1	CMC	Oven	3.15 ± 0.04	88.9 ± 0.06
		2		3.43 ± 0.06	95.0 ± 0.03
		3		3.29 ± 0.06	45.8 ± 0.04
		4		3.17 ± 0.03	19.0 ± 0.05
IR-1		CMC	IR	3.14 ± 0.02	63.2 ± 0.03
		2		3.39 ± 0.03	120.9 ± 0.04
		3		3.28 ± 0.02	70.9 ± 0.02
		4		3.15 ± 0.04	34.1 ± 0.03
OIR-1		CMC	Oven + IR	3.33 ± 0.06	33.3 ± 0.03
		2		3.45 ± 0.04	90.3 ± 0.02
		3		3.38 ± 0.03	44.6 ± 0.03
		4		3.33 ± 0.04	18.0 ± 0.06
OD-2	2	CMC	Oven	3.12 ± 0.02	19.5 ± 0.02
		2		3.38 ± 0.03	36.1 ± 0.05
		3		3.22 ± 0.04	32.1 ± 0.06
		4		3.11 ± 0.03	12.5 ± 0.03
IR-2		CMC	IR	3.09 ± 0.06	70.6 ± 0.03
		2		3.31 ± 0.04	71.9 ± 0.04
		3		3.21 ± 0.03	50.0 ± 0.05
		4		3.11 ± 0.06	34.0 ± 0.03
OIR-2		CMC	Oven + IR	3.31 ± 0.04	20.0 ± 0.06
		2		3.43 ± 0.03	24.4 ± 0.02
		3		3.37 ± 0.06	17.2 ± 0.06
		4		3.31 ± 0.03	7.1 ± 0.05
